# Outcome of survivors of COVID-19 in the intermediate phase of recovery: A case report

**DOI:** 10.4102/sajp.v78i1.1751

**Published:** 2022-03-31

**Authors:** Marelee Fourie, Heleen van Aswegen

**Affiliations:** 1 Michele Carr Physiotherapists, Wits Donald Gordon Medical Centre, Johannesburg, South Africa; 2 Department of Physiotherapy, Faculty of Health Sciences, University of the Witwatersrand, Johannesburg, South Africa

**Keywords:** COVID-19, inspiratory muscle training, exercise therapy, six-minute walk test, peak expiratory flow

## Abstract

**Introduction:**

Coronavirus disease 2019 (COVID-19) is a viral respiratory disease and is associated with significant morbidity in the intermediate and chronic phases of recovery from the disease. The health benefits of respiratory and extremity muscle strengthening exercise therapy are well-described for those with cardiac failure and interstitial lung disease and are suggested to improve functional ability for patients recovering from COVID-19. The aim of this case report is to share the effects of standard physiotherapy management on exercise endurance, respiratory function and return to work, implemented for patients with COVID-19 in the intermediate phase of their recovery.

**Patient presentation:**

Two cases of COVID-19 were admitted to a private healthcare facility in Johannesburg. They presented with shortness of breath and decreased endurance. One had COVID-19 myocarditis and the other chronic post-COVID-19 organising pneumonia with pulmonary fibrosis.

**Management and outcome:**

Both patients were admitted to ICU, provided oxygen therapy and supportive care as well as physiotherapy management in hospital and after hospital discharge. Physiotherapy management included inspiratory muscle training therapy, and cardiovascular and resistance exercise therapy. Improvements in peak expiratory flow rate and six-minute walk distance were observed for both cases at 6- and 7-months follow-up, respectively.

**Conclusion:**

Our case report illustrates the value of ongoing physiotherapy management, utilising progressive exercise therapy prescription, to aid the return to optimal functioning for survivors of COVID-19 in the intermediate phase of their recovery.

## Introduction

An outbreak of severe acute respiratory syndrome coronavirus-2 (SARS-CoV-2) in 2019 led to the spread of coronavirus disease 2019 (COVID-19) into a global pandemic with millions of people affected in all countries (Barker-Davies et al. 2020). The severity of COVID-19 ranges from very mild symptoms that clear up within days to severe symptoms that require admission to an intensive care unit (ICU) and the need for mechanical ventilation if respiratory or cardiac failure develops (Barker-Davies et al. 2020; Dhakal et al. 2020). Chest tightness and shortness of breath (SOB), muscle soreness, wasting and weakness, including impairments in physical and respiratory functioning are known to develop as a result of elevated inflammatory markers and disease severity (Hosey & Needham 2020; Zhu et al. 2020). Coronavirus disease 2019 is a viral respiratory disease and is associated with significant morbidity in the intermediate (3–6 months) and chronic phases (≥ 12 months) of recovery due to its impact on cognitive, physical, psychological and respiratory functions (Barker-Davies et al. 2020; Hosey & Needham 2020). Symptoms of headache, fatigue and dyspnoea amongst patients with increasing age, body mass index and the female sex are typical of long COVID-19 (Sudre et al. 2021). Physiotherapy rehabilitation of patients with COVID-19 commences in the ICU setting and progression of rehabilitation should continue after hospital discharge to ensure optimal recovery (Barker-Davies et al. 2020; Hosey & Needham 2020). The health benefits of respiratory and extremity muscle strengthening exercise therapy are well-described for those with cardiac failure and interstitial lung disease and these benefits include an improvement in the functional ability of patients recovering from COVID-19 (Dallas et al. 2021; Reina-Gutierrez et al. 2021). The aim of this case report is to share the effects of standard physiotherapy management (utilising chest clearance techniques and exercise prescription) on exercise endurance, respiratory function and return to work, implemented for patients with COVID-19 in the intermediate phase of their recovery.

## Patient presentation

Both patients presented in this report provided informed consent for their anonymised information to be shared.

Mrs K, a 48-year-old female with known hyperthyroidism, developed mild symptoms of COVID-19 in August 2020. She managed her symptoms through convalescence at home. In October 2020, she was admitted to a private healthcare facility in Johannesburg with mild SOB limiting daily activities and decreased endurance. On examination, her left ventricular ejection fraction was 37%, she required 2 L/min oxygen therapy to manage her SOB and was diagnosed with COVID-19 myocarditis.

Mr M is a 48-year-old male with known asthma, hypertension, diabetes mellitus, hypothyroidism, chronic sinusitis and gastric reflux. He contracted COVID-19 which resulted in three separate private sector hospitalisations with intubation during the first hospitalisation. He was transferred to this private healthcare facility in Johannesburg by the end of August 2020. On examination, he presented with extreme SOB with minimal activity (Medical Research Council Dyspnoea scale level 5), required 45 L/min high-flow oxygen therapy and used continuous positive airway pressure (CPAP) therapy at night. Mr M was diagnosed with chronic post-COVID-19 organising pneumonia and subsequent pulmonary fibrosis.

## Management and outcome

Mrs K underwent an angiogram, was admitted to ICU (not intubated), received supportive care and participated in four physiotherapy treatment sessions. She responded well to therapy received and was discharged home after 4 days. One week later, she commenced outpatient physiotherapy which consisted of a needs-assessment, inspiratory muscle training (IMT) therapy (level 2 resistance 30 breaths bi-daily), daily walking programme (guided by 50% – 60% age predicted maximal heart rate (HR_max_)) and resistance exercise therapy (twice weekly).

**FIGURE 1:**
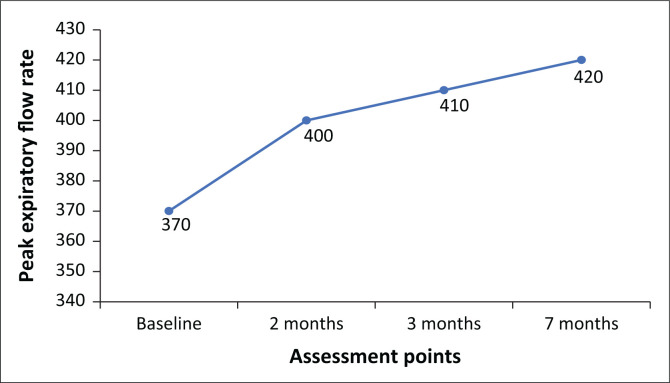
Changes in peak expiratory flow rate for Mrs K over a 7-month follow-up period.

**FIGURE 2:**
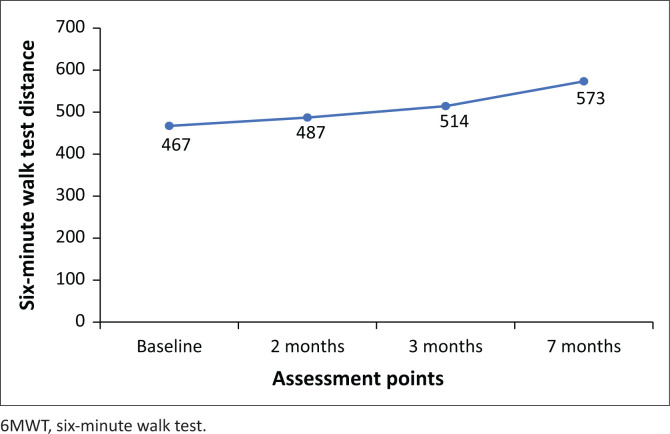
Changes in distance walked by Mrs K over a 7-month follow-up period.

Progression of IMT (level 5 resistance, 30 breaths bi-daily) and walking programme (60% – 70% age-predicted HR_max_) occurred monthly as tolerated. Improvements in peak expiratory flow rate (PEFR) and six-minute walk test (6MWT) distance were observed over the 7-months period (Figure 1 and Figure 2). At final assessment in May 2021, she had a normal PEFR (420 L/min age-and-gender predicted), normal ejection fraction and achieved 88% predicted 6MWT distance (651 m age-and-gender predicted). She had returned to work and was subsequently discharged from physiotherapy.

Mr M was admitted to ICU and provided with non-invasive ventilation. He received supportive care and 22 in-hospital physiotherapy treatments during which IMT therapy was commenced 3 weeks prior to discharge (level 4, 30 breaths bi-daily). He was discharged after 52 days and outpatient physiotherapy was commenced in November 2020, consisting of a needs-assessment, ongoing IMT therapy, daily walking and cycling programme (60% – 70% age-predicted HR_max_) and resistance exercise therapy (twice weekly). Outpatient management included chest clearance therapy and functional training activities. Changes in PEFR and 6MWT distance were observed over the 6-months follow-up period (Table 1). At final assessment in May 2021, he achieved 80% of age-predicted 6MWT distance and 98% predicted PEFR off oxygen. Forced vital capacity improved from 47% to 69%. He still used low-flow oxygen therapy intermittently and slept with CPAP at night. He was considered fit for return to work and discharged from physiotherapy.

**TABLE 1:** Changes in peak expiratory flow rate and six-minute walk test distance for Mr M over a 6-month follow-up period.

Assessment points after hospital discharge	Peak expiratory flow (L/min) (Age-predicted normal = 600 L/min)		6MWT distance (meters) (Age-predicted normal = 752 m)	
	Actual	% Predicted	Actual	% Predicted
Baseline	650	108	548.0	73
2 months	570	95	544.5	72
6 months	590	98	603.0	80

6MWT, six-minute walk test.

## Conclusion

Persistent symptoms of fatigue and muscle weakness are experienced by some survivors of COVID-19 during and long after recovery (Disser et al. 2020; Sudre et al. 2021). Severe acute respiratory syndrome coronavirus-2 uses angiotensin-converting enzyme-2 (ACE2) receptors to enter human cells. Many organs, including skeletal muscle smooth cells and cardiac cells, express considerable amounts of ACE2 genes for organ protection, which becomes ineffective after infiltration by SARS-CoV-2 (Dhakal et al. 2020; Disser et al. 2021). This could possibly explain the persistent symptoms of fatigue and weakness reported. Researchers from China reported that those with acute COVID-19 who required oxygen therapy achieved 85% – 88% predicted 6MWT distance at 6 months (Huang et al. 2021), which was similar to that of Mrs K (Huang et al. 2021). Mr M, however, had lower exercise capacity at 6 months. Altered mechanical properties of his airways because of resultant pulmonary fibrosis and more severe COVID-19 infection could explain his lower exercise capacity (Plantier et al. 2018). Inspiratory muscle training reduces dyspnoea and improves exercise endurance more in less fit healthy individuals than those who are high-performing athletes and in those with chronic pulmonary diseases (Illi et al. 2012; Langer et al. 2018; Reina-Gutierrez et al. 2021), which could explain why Mrs K and Mr M benefited from IMT training in the intermediate phase of their recovery from COVID-19. Individualised assessment and exercise prescription for patients recovering from COVID-19 are essential (Barker-Davies et al. 2020; World Physiotherapy 2021). This approach was adopted in the management of the two cases presented, with progression of exercise therapy implemented only when tolerated. Changes in 6MWT distances observed were 106 m and 55 m, respectively, which are greater than the minimum importance difference of 25 m reported for patients with chronic pulmonary diseases (Holland et al. 2010). Our case report illustrates the value of ongoing physiotherapy rehabilitation, utilising progressive exercise therapy prescription, for survivors of COVID-19 in the intermediate phase of recovery, in aiding their return to optimal functioning.
